# Immune Infiltration and Prostate Cancer

**DOI:** 10.3389/fonc.2015.00128

**Published:** 2015-07-08

**Authors:** Amy Strasner, Michael Karin

**Affiliations:** ^1^Laboratory of Gene Regulation and Signal Transduction, Department of Pharmacology, University of California San Diego School of Medicine, La Jolla, CA, USA; ^2^Laboratory of Gene Regulation and Signal Transduction, Department of Pathology, University of California San Diego School of Medicine, La Jolla, CA, USA

**Keywords:** prostate cancer, immune cells, inflammation, immunotherapy, B cells

## Abstract

It is becoming increasingly clear that inflammation influences prostate cancer (PCa) development and that immune cells are among the primary drivers of this effect. This information has launched numerous clinical trials testing immunotherapy drugs in PCa patients. The results of these studies are promising but have yet to generate a complete response. Importantly, the precise immune profile that determines clinical outcome remains unresolved. Individual immune cell types are divided into various functional subsets whose effects on tumor development may differ depending on their particular phenotype and functional status, which is often shaped by the tumor microenvironment. Thus, this review aims to examine the current knowledge regarding the role of inflammation and specific immune cell types in mediating PCa progression to assist in directing and optimizing immunotherapy targets, regimens, and responses and to uncover areas in which further research is needed. Finally, a summary of ongoing immunotherapy clinical trials in PCa is provided.

## Introduction

Prostate cancer (PCa) is the most common non-cutaneous cancer and second leading cancer-related cause of death of men in the United States. (1). Localized PCa can be controlled with surgery or radiation over 80% of the time. If prostate tumors are metastatic at presentation, neither surgery nor radiation will confer significant clinical benefit and androgen deprivation therapy (ADT) represents the sole therapeutic option for such patients. Unfortunately, resistance to hormonal targeting develops in most patients within 2 years, a state that is termed metastatic castration-resistant PCa (mCRPC) and is virtually untreatable (2, 3). Until recently, Docetaxel was the lone accepted remedy for mCRPC and was associated with a 2- to 3-month enhancement of survival (4–6). The advent of newer chemotherapeutic agents such as cabazitaxel and approval of next generation drugs abiraterone and enzalutamide have improved upon this effect (7–10), yet resistance ultimately ensues, possibly via mutations in the androgen receptor (*AR*) gene (11).

Relative to other forms of cancer, the discovery of valuable predictive markers of disease progression has been particularly challenging for PCa. Genetic markers capable of identifying clinically aggressive disease remain elusive (12, 13). In addition, PCa is unique among solid tumors in that imaging techniques are insufficient for definitive identification and diagnosis of malignancy (12, 14). Instead, the pre-biopsy serum prostate specific antigen (PSA) value is used as an indicator of potential underlying disease, but the utility of this screening method has been intensely scrutinized (15). In fact, the United States Preventative Services Task Force (USPSTF) recently recommended against routine PSA screening (16). Importantly, the ramifications of this ruling may be an increase in the proportion of men diagnosed at later stages of PCa (17). Thus, the identification of novel therapeutic targets for and biomarkers of mCRPC is severely needed.

A multitude of studies investigating alternative PCa treatment interventions or predictive markers have focused on components of the inflammatory response, with promising results. A relationship between inflammation and cancer was first proposed by Virchow in 1863 (18) and chronic inflammation is now well accepted as a driving force behind many malignancies, such as gastric, colon, esophageal and lung cancers, and hepatocellular carcinoma (19). While the role of the immune system in PCa is less clear, it has been implicated both as a possible etiologic agent and a tumor promoter. A recent report from the REDUCE study suggesting that aspirin and/or NSAID use decreased risk of PCa in men with a negative biopsy supports this notion (20). This review will explore the evidence regarding immune infiltration as a potential mediator and indicator of aggressive PCa, as well as a target for PCa therapeutics. Because this topic has been comprehensively evaluated elsewhere (21–26), only recent literature will be addressed here.

## Prostatitis and Prostate Cancer: The Evidence

As with many other cancers, PCa arises from accumulation of acquired genetic and epigenetic alterations. Currently, age, ethnicity, and family history are the only known risk factors for development of prostate neoplasia. These genetic traits may partially explain the geographical variance in PCa incidence and mortality, with rates in Southeast and East Asia being much lower than those in the United States and Western Europe (22, 27). By contrast, the elevated rate of PCa observed in Chinese and Japanese men within one generation of relocation to the West indicates that environmental factors, particularly lifestyle factors such as diet, also contribute to the emergence of PCa (28). One likely link between environmental stimuli and PCa occurrence is chronic inflammation. Support for this hypothesis comes from reports of focal atrophic lesions in the prostate containing a relatively high percentage of proliferating epithelial cells and acute or chronic inflammatory infiltrate. These areas, designated proliferative inflammatory atrophy (PIA), comprise an extensive portion of the peripheral zone of the prostate, the primary site where PCa originates, and have been suggested to be precursors of prostatic intraepithelial neoplastic (PIN) lesions (22, 24, 29), although one recent retrospective epidemiological study refuted this concept (30). Additional evidence comes from a recent meta-analysis assessing 20 case–control studies conducted between 1990 and 2012 that revealed a significant positive relationship between self-reported prostatitis and PCa. However, the authors were unable to demonstrate a causal link between the two due to the absence of cohort studies in this analysis (31). Numerous other epidemiological investigations have examined the connection between prostatitis and subsequent PCa risk and while early studies (32–35) described an elevated risk of malignancy or recurrence due to presence of inflammation, subsequent studies have produced conflicting results (29, 30, 36–40). In addition to differences in study populations and potential selection and other forms of bias, plausible explanations for these inconsistencies include tissue sample size and composition, definition and measurement of inflammation, tissue regions assessed, and categories of prostatitis examined. In order to obtain a more accurate reflection of the relationship between prostatitis and PCa, improved standardization of these measurements, incorporation of asymptomatic inflammatory prostatitis, and a prospective design in future epidemiological studies are needed. Importantly, many investigations defined inflammation with discrete variables, such as present or absent or severe, moderate or mild rather than with a detailed analysis of the cellular composition and distribution of the infiltrate. Although this classification system may be obligatory for certain statistical analyses, it is a major limitation of these investigations and may be masking true associations that exist. Furthermore, these studies may benefit from experimental research designed to provide insight regarding the kinetics, distribution, type, and components of inflammation that are important for prostate tumorigenesis.

Despite the abundance of data evaluating the connection between prostatitis and PCa, the sources of prostatic inflammation have yet to be fully elucidated. Proposed causes can be divided into three broad categories: (1) microorganism induced, including *E. coli*, *Propionibacterium acnes*, and those associated with urine reflux and sexually transmitted diseases, (2) non-microorganism induced, such as uric acid crystals formed during urine reflux, estrogen, dietary factors, especially heterocyclic amines (HCA) 2-amino-1-methyl-6-phenylimidazo[4,5-b] pyridine (PhIP) generated from cooking meats at high temperatures (41), obesity, and physical trauma such as corpora amylacea (23–25, 42–44), and (3) treatment- or tumor-elicited inflammation (45, 46).

Some of these hypotheses were tested using mouse models. For example, Shinohara et al. reported that intraurethral inoculation of mice with a prostatectomy-derived strain of *P. acnes* elicited prolonged and lobe-specific severe acute and chronic inflammation that was associated with increased proliferation and reduced Nkx3.1 and AR expression. Inflammatory infiltrate consisted of luminal neutrophils and stromal mononuclear cells (44). Similar observations were noted in experiments using *E. coli*. Introduction of this bacterium into mouse urogenital tracts provoked a dramatic decline in Nkx3.1 and AR expression and a corresponding enhancement of proliferation as well as the basal cell marker p63. These changes were pursuant to an early influx of neutrophils into prostatic lumen and stroma followed by a surge in stromal mononuclear cells and later a marked increase in plasma cells (43). Upregulation of pro-inflammatory genes, including IL-6 and COX-2, was also detected using a comparable system (42). The effects of these events on PCa pathogenesis were examined by combining bacteria CP9-induced prostatitis and a mouse PCa model and revealed that tissue damage resulting from acute prostatitis facilitates accumulation of stromal α-smooth muscle actin-positive myofibroblasts and the conversion of basal cells into luminal cells, which accelerates PCa progression (47).

Non-bacterial inflammatory stimuli were also evaluated with respect to PCa development. A novel model of prostatitis involving ubiquitous overexpression of aromatase (AROM^+^) in mice exhibited early and persistent prostatic mast cell infiltration with subsequent accumulation of neutrophils, T lymphocytes, and macrophages, as well as increased expression of numerous chemokines. This chronic inflammation preceded formation of PIN lesions containing and surrounded by immune cells (48). Finally, Birbach et al. expressed a constitutively active form of the inflammatory mediator IκB kinase 2 in prostate epithelium of mice harboring a heterozygous *Pten* deletion and detected a robust inflammatory response, primarily encompassing neutrophils and macrophages in the prostate, that was accompanied by increased tumor size due to enhanced epithelial and stromal proliferation, generation of cribiform structures and stromal remodeling, but no invasion or metastasis (49).

Together, these data corroborate the ability of inflammatory stimuli to generate an immune response that modulates the local environment into one that is conducive to malignant transformation and progression and implicate chronic inflammation as a causative factor in PCa. More studies combining prostatitis and murine PCa models are needed to confirm these effects and determine the mechanisms by which these agents promote disease. Of course, these results also must be confirmed in humans.

## Immune Cells and Prostate Cancer: Friends or Foes?

As previously described, epidemiological studies exploring the relationship between prostatitis and PCa development may be limited by the absence of characterization of cellular phenotypes displayed by tumor-infiltrating leukocytes (TIL). However, many studies assessing the association between specific immune cells and PCa are now available and those conducted between 2008 and 2014 are summarized according to cell type in Table 1. Most of these investigated CD3^+^, CD4^+^, or CD8^+^ cells in prostatectomy or biopsy tissue of PCa patients and found such cells to be pro-tumorigenic. For example, a cross-sectional study of lymphocyte infiltration in tumor tissue microarrays from patients with PSA recurrence reported that extremely high or low CD3^+^ cell counts were correlated with reduced PSA recurrence-free survival (RFS) (50). Importantly, CD3 was the only T-cell marker included in this study and no evaluations of T-cell subsets were performed. Thus, the variation in clinical outcomes across CD3^+^ cell quartiles may at least partially reflect differences in T-cell populations. In fact, the authors mentioned unpublished data indicating that higher numbers of regulatory T-cells (T_reg_) were associated with more advanced tumor stage and PSA-RFS. A likely assumption is that the CD3^+^ cells whose low numbers conferred poor prognoses were protective in nature. Thus, it is critical to determine not only the cell type but also the subtype associated with different outcomes in order to better direct therapeutic efforts. By contrast, the spontaneous TRAMP mouse PCa model was used to illustrate that the trafficking of a naturally arising clonally expanded PCa histone H4-specific population of CD44^+^CD8^+^ T-cells to prostate tissue lessened tumor burden but failed to improve survival (51). Several possible explanations for the lack of survival impact exist. First, it may be that the anti-tumorigenic CTL were initially successful in eliminating tumor cells but were eventually rendered inactive by a suppressive tumor microenvironment. Alternatively, it is conceivable that the antigen-specific destruction of H4-expressing tumor cells selected for less immunogenic and possibly more adept H4-negative tumor cells, or instead, that this response exerted pressure on cancer cells to downregulate MHC or undergo immunoediting of tumor antigens and thereby escape immune recognition and destruction. In line with the former hypothesis, several investigations demonstrated that tumor-infiltrating CTL expressed PD-1, one of several indicators of T-cell exhaustion, in PCa patients (52, 53). This is an essential consideration for immunotherapeutic efforts and emphasizes the importance of evaluating both the cellular subtype and functional status when assessing TIL.

**Table 1:** **Immune cells and prostate cancer**.

**T-cells (CD4^+^, CD8^+^, T_H_17)**	
Fujii (80)	Neutral; no difference in number of CD3^+^ cells in glandular hyperplasia, PIN, or adenocarcinoma tissues.
Yuan (81)	Positive; lymphocyte aggregates found almost exclusively (95.5%) in normal appearing or pre-invasive epithelial structures, many physically attached to basal cell layers that were often focally disrupted or substantially attenuated. Significantly, fewer aggregates were seen within invasive tissue. Epithelial cells overlaying immune cells attached to focal disruptions displayed increased stem cell markers, invasive morphology, and proliferation. CTL was the predominant infiltrating cell type, although mast and NK cells were also present.
Flammiger (50)	Positive for high or low CD3^+^ cell numbers and PSA recurrence-free survival. CD3 T-cells were also higher in ERG positive tumors than ERG negative tumors. CD3 T-cells were primarily found in stroma and rarely in prostate epithelium.
Valdman (58)	Positive for cancer progression; CD4^+^ and CD8^+^ cells were increased in cancer vs. non-atrophic benign tissue, but did not correlate with Gleason Score.
Gannon (55)	Positive for elevated CD3^+^ cells and invasion in control group; ADT increased relative abundance of CD3^+^ and CD8^+^ lymphocytes vs. control group. No correlations with clinical parameters or recurrence were observed in ADT group.
Sfanos (53)	N/A: substantial degree of prostate specific CD8^+^ T-cell clonality in prostate cancer tissue but not peripheral blood; however, PD-1 was relatively upregulated on CD8^+^ T-cells infiltrating prostate gland in men with cancer. Clonality was patient-specific.
Ebelt (52)	N/A: previously described pronounced clusters of CD3^+^ cells, predominantly CD4^+^, formed adjacent to tumor islets. Now report the presence of PD-1^+^ and B7-H1^+^ cells in these clusters and in BPH, but not in carcinoma area or healthy tissues.
Ellem (48)	Positive: CD3^+^ cells increased in the AROM^+^ mouse model by 40 weeks and PIN lesions reminiscent of those in humans developed following chronic inflammation. A large amount of the inflammatory infiltrate was seen in lumen and stroma surrounding lesions and in lesions themselves.
Sfanos (82)	Negative: numerically, most abundant CD4^+^ cell subset was IFNγ producing T_H_1 cells. CD4^+^ prostate infiltrating lymphocytes were skewed toward either a regulatory T (Foxp3^+^) or T_H_17 subtype. T_H_17 cells showed an inverse correlation with Gleason Score.
Savage (51)	Negative: a naturally arising, clonally expanded population of CD8^+^CD44^+^ cells that recognize Histone 4 and are prostate cancer specific in TRAMP mice. These cells traffic to the prostate, proliferate, and reduce tumor burden, but have no effect on overall survival.
**B cells**
Woo (70)	Neutral for clinical parameters, but higher in malignant vs. benign for overall cohort and high risk and recurrent patients.
Fujii (80)	Negative; frequency of B cells in benign glands was 59%. This was significantly reduced in PIN and adenocarcinoma.
Flammiger (50)	Neutral; number of CD20^+^ B cells not associated with tumor stage, lymph node status, Gleason Score, preoperative PSA, or PSA recurrence-free survival.
Ammirante (45)	Positive; LTb expressing B cells infiltrate regressing tumors in response to castration-induced cell death and CXCL13 production and mediate progression to CRPC. Tumor-infiltrating B cells peaked at 1 week and receded by 2 weeks post-castration.
Gannon (55)	Neutral; no change in abundance of CD20^+^ cells in ADT vs. control group.
Ellem (48)	Neutral; no change in CD45R^+^ B cells in prostate in AROM^+^ mouse model.
Shalapour et al. (69)	Positive; Immunosuppressive IgA^+^IL-10^+^PD-L1^+^ plasmocytes interfere with the immunogenic cell death mediated CTL response to low-dose oxaliplatin.
**Macrophages**
Lanciotti (54)	Positive for M2 macrophages; 36.6% of patients had higher prevalence of M1 (CD68^+^) macrophages and 63.4% had higher abundance of M2 (CD163^+^) macrophages. M1 occurred more frequently in organ confined disease and M2 was associated with more advanced disease. M1:M2 correlated with disease stage while M2 phenotype was related to extracapsular extension. No correlation between M1:M2 ratio and recurrence.
Fujii (80)	Positive; CD204^+^: CD68^+^ macrophage ratio increased in PIN and adenocarcinoma compared to benign tissue.
Gollapudi (83)	Positive for cancer progression; mean CD68^+^ tumor-associated macrophage (TAM) higher in cancer cores than PIN, which was higher than benign. Mean TAM higher in Gleason grade 4 than grade 3. No association in mean TAM in cancer cores and age, PSA, or prostate cancer recurrence following radical prostatectomy.
Nonomura (56)	Positive; patients with higher stage or Gleason Score prostate cancer had higher CD68^+^ TAM counts. Patients with PSA failure had significantly higher TAM counts than those without. Recurrence-free survival was significantly lower in patients with high TAM counts than those with low TAM counts. TAM count was determined to be a significant prognostic factor in addition to PSA, Gleason Score, extraprostatic extension, lymph node metastasis, and distant metastasis.
Gannon (55)	Positive; ADT increases the abundance of CD68^+^ macrophages. This elevated relative abundance of CD68^+^ cells favored prostate cancer progression and recurrence in control group. No correlations with clinical parameters or recurrence were observed in the ADT group.
Craig (84)	Positive; coinoculation of athymice nude mice with PC3^+^U937^IL-4^ or PC3^+^U937 decreased time to critical tumor mass and increased angiogenesis and CCL2 expression compared to PC3 alone.
Ellem (48)	Positive: F4/80^+^ cells increased in the AROM^+^ mouse model by 40 wks and PIN lesions reminiscent of those in humans developed following chronic inflammation.
**Mast cells**
Johansson (63)	Positive for peritumoral mast cells but negative for intratumoral mast cells: mast cells are independent prognostic variables of prostate cancer progression, but peritumoral and intratumoral mast cells have differential effects. In animals, mast cells stimulate angiogenic activity and growth to drive disease progression, possibly via VEGF expression. Peritumoral but not intratumoral mast cells in humans correlated with poor prognosis. Intratumoral mast cells were negatively associated with metastasis, Gleason score, tumor stage, peritumoral vessel density, and proliferation and were associated with a good clinical outcome. Mast cells were also recruited to relapsing prostate tumors following castration therapy in rats.
Ellem (48)	Positive: mast cell numbers significantly increased throughout tissue immediately after puberty and persisted throughout life in the AROM^+^ mouse model.
Pittoni (64)	Positive and negative: mast cells (c-Kit^+^FcεRI^+^CD45^+^CD11b^−^ for FACS and toluidine blue for IHC) increase in prostate tissue upon progression from PIN to well-differentiated (WD) tumors in response to SCF production, but are absent in poorly differentiated (PD) tumors, which lack SCF expression. Pharmacological reduction of mast cells suppressed or prevented WD but not PD tumor growth via MMP-9 production. PD exhibited autocrine production of MMP-9. This pattern was recapitulated in human prostate cancer tissue. Paradoxically, targeting mast cells in TRAMP mice promoted development of aggressive c-Kit^+^ neuroendocrine tumors.
Fleischman (85)	Negative: strong association between high-mast cell (c-Kit^+^) densities and favorable tumor characteristics in TMA from hormone-naïve patients. PSA-RFS significantly declined for patients with low-mast cell density and was worst for those who completely lacked mast cells.
**Immunosuppressive cells (T_regs_, MDSCs)**	
Valdman (58)	Positive for progression but not clinical outcomes: infiltration of Foxp3^+^ cells was increased in cancer compared to non-atrophic benign tissue but did not correlate with Gleason score.
Ebelt (52)	Positive for progression but not clinical outcomes: Foxp3^+^ cells were present in previously described clusters of CD4^+^ lymphocytes adjacent to tumor cells but were virtually absent in carcinoma area. Collectively, Foxp3^+^ cells were more frequent in cancer than BPH or healthy tissue, but were not correlated with Gleason score or TNM.
Garcia (60)	Positive: expansion of immunosuppressive (iNOS, arginase) CD11b^+^Gr1^+^ MDSC in prostate immediately following *Pten* deletion in a murine prostate cancer model. This was particularly apparent at invasive carcinoma stage and was likely due to upregulation of epithelial cell-derived CSF1 and IL1β.
DiMitri (59)	Positive: massive infiltration of Gr1^+^CD11b^+^ cells at onset of senescence induced by complete PTEN loss (with or without Docetaxel). These cells expressed IL1-RA and opposed IL-1α-induced senescence, thereby promoting tumor progression. This phenomenon was also observed in oncogene-induced tumor models. Human prostate cancer patients whose tumors express high levels of IL1-RA did not respond to Docetaxel and had shorter DFS.
Idorn (61)	Positive: both Docetaxel and untreated patients exhibited high frequencies of myeloid-derived suppressor cells (MDSC) (HLA-DR^low/neg^Lin^−^CD11b^+^CD33^+^) as compared to healthy donors. MDSC iNOS expression and frequency of circulating T_reg_ (CD3^+^CD4^+^CD25^hi^CD127^low^) were significantly higher in both groups of prostate cancer patients than healthy controls. MDSC levels were significantly higher in patients with three negative prognostic factors (elevated LDH, AP, PSA, anemia) vs. ≤1.
Derhovanessian (62)	Positive for T_H_17 cells but not T_regs_: prevaccination frequency of IL-17 cells, but not T_regs_ predicted time to progression in hormone-resistant prostate cancer patients. Frequency of IL-17^+^ cells lacking CCR4 expression was indicative of poor prognosis in these individuals.
**Neutrophils**
Sonpavde (86)	Positive: high neutrophil: lymphocyte ratio (NLR) associated with independent poor prognostic impact in post-docetaxel patients with mCRPC.
Fujita (87)	Negative: neutrophil count was found to be an independent predictor of prostate cancer in biopsies of Japanese men with elevated PSA levels or abnormal DRE. An elevated neutrophil count may predict a benign prostate biopsy. A low neutrophil count and elevated PSA level may be good indicators for biopsy.
Sümbül (88)	Neutral: NLR was found to be effective in predicting PSA but not clinical response to docetaxel + prednisone therapy in CRPC patients.
Templeton (89)	Positive: NLR > 3 was one of a number of variables in a prognostic risk score in men with mCRPC treated with Docetaxel.
Nuhn (90)	Positive: mCRPC patients with low pretreatment NLR (≤3) had significantly longer overall survival than those with high NLR following first-line Docetaxel therapy.
Shafique (91)	Neutral: NLR not associated with survival or risk of death in patients with prostate cancer from Glasgow Inflammation Outcome Study. By contrast, mGPS, a combination of C-reactive protein and albumin, predicted poorer 5-year overall survival and relative survival independent of age, socioeconomic status, disease grade, and NLR. Elevated mGPS also had sig. association with excess risk of death among aggressive, clinically sig. prostate cancer (Gleason 8–10).
Sadeghi (92)	Negative: neutropenic [absolute neutrophil count) (ANC) <1.5 × 10^9^/L] African-American patients had significantly higher clinical stages and pathologic Gleason scores at radical prostatectomy. ANC was sig. predictive of high tumor grade.

Another cell type that has received much attention and shown relatively consistent results in the context of PCa progression is the tumor-associated macrophage (TAM). All studies analyzed demonstrated a pro-tumorigenic effect of TAM, although one report (54) specified that this was true only for M2 type macrophages. However, only a fraction of the literature displayed a link between TAM and recurrence (55, 56). In particular, Nonomura et al. (56) determined TAM count to be a significant prognostic factor of clinical outcome in PCa patients. A second study that found TAM to be related to recurrence indicated this to be true only in the control but not ADT treated group. Thus, TAM count may be a good biomarker for disease progression, but further research is needed to identify the specific subset of macrophages and PCa patients for which this is applicable. TAM may also represent a viable therapeutic target for PCa, as demonstrated by Escamilla et al., who showed a reduction in castration-induced recruitment of pro-tumorigenic TAM and delayed emergence of CRPC in a mouse Myc-CaP PCa model following CSF1R inhibition (57).

As expected, presence of immunosuppressive immune cells, such as T_reg_ (52, 58), myeloid derived suppressor cells (MDSC) (59–61), and according to one report, T_H_17 cells (62), in prostate tissue was suggestive of more aggressive disease, but surprisingly few studies were able to equate this with clinical prognosis. Neutrophils and mast cells have also been fairly well studied, but investigations involving both cell types have generated conflicting results concerning their influence on PCa. One source of contention may concern the area of tissue evaluated. This point is nicely illustrated by Johannson et al. (63), who implicated peritumoral but not intratumoral mast cells as independent prognostic variables of PCa progression. In addition, work from Pittoni et al. (64) emphasized that care must be taken to evaluate potential negative outcomes when manipulating immune cell populations with therapies. In the TRAMP model of murine PCa, targeting mast cells prevented growth of well-differentiated tumors but simultaneously stimulated progression of more aggressive, neuroendocrine type tumors. Similarly, Tang et al. observed a late IL-2-induced expansion of T_reg_ following castration and immunization that negated the initial anti-tumorigenic CD8^+^ T-cell response (65).

Relative to other immune cells, B cells have been generally overlooked in terms of PCa. However, the importance of these cells in PCa progression was established in a landmark and subsequent papers (45, 66, 67) using subcutaneous Myc-CaP and spontaneous TRAMP mouse PCa models to show that castration-induced cell death and resulting hypoxia stimulated CXCL13-mediated infiltration of lymphotoxin β (LTb)-expressing B lymphocytes into regressing tumors. These cells subsequently activated IKKα, STAT3 and BMI1 in cancer cells to promote CRPC progression. Notably, interference with any aspect of this pathway delayed emergence of hormone-resistant tumors. These initial findings were extended to reveal that the identical mechanism operates in androgen-dependent regeneration of normal prostate progenitor cells (67). Interestingly, an independent investigation reported significant reductions in human and murine PCa tumor growth following combination therapy with HIF-1α and phospho-STAT3 inhibitors (68). Together, these data suggest that the B cell response to tissue injury or death following androgen depletion drives restorative or aberrant proliferation in the prostate. In addition, a recent report implicates immunosuppressive IgA^+^PD-L1^+^IL-10^+^ plasma cells in impeding response to low-dose chemotherapy in a T-cell-dependent manner (69). The presence of CD20^+^ B cells has been documented in human PCa as well, and the density of these cells was shown to be higher in malignant than benign tissue (70). Furthermore, a case study of an advanced PCa patient treated with the B cell depleting antibody Rituximab produced fairly encouraging results (71). A Phase 0 clinical trial evaluating Rituximab as neoadjuvant therapy in patients prior to radical prostatectomy is currently ongoing. Thus, although research is still in preliminary stages, B cells may represent an emerging target for PCa immunotherapeutics. However, it is likely that a damage response (45, 67) and/or an immunogenic stimulus (69) is needed to make these cells relevant in prostate carcinogenesis or that only particular subsets, such as those expressing LTβ, or IgA are crucial.

## Prostate Cancer and Immunotherapy

The emergence of immune cells as important mediators of PCa progression has spawned a multitude of clinical trials targeting these cells via different approaches, including therapeutic vaccines, checkpoint inhibitors/immune modulators, adoptive T-cell therapy, and monoclonal antibodies. In theory, PCa should be particularly conducive to this type of treatment due to its long latency period. However, because PCa is commonly diagnosed late in life, therapeutic vaccines may not be as efficacious as desired due to the age-related decline in immune responsiveness. Despite this fact, Sipuleucel-T was the first immunotherapy approved by the FDA for PCa and has shown an overall survival advantage in mCRPC patients, although no effect on progression-free survival was noted (72). The lack of short-term response observed in this study may at least partially be partially attributed to the fact that immunotherapeutic agents are not directly cytotoxic, as are more standard treatments. Due to space limitations, individual studies will not be discussed in detail here but are summarized in Table 2 (2, 72–77). One important message from the combined studies is that, while extremely promising, these immunotherapeutic methodologies still need to be optimized as no individual treatment has yet elicited a complete response. Complete and durable responses will likely require combinations of current immunotherapy and/or more standard approaches, such as chemotherapy, radiation, or ADT, which induce an immunogenic stimulus or enhance T-cell responses. In addition, ideal target patient populations and treatment protocols still need to be identified. For example, a study combining PROSTVAC and ipilimumab discovered that patients with lower PD-1^+^Tim-3^NEG^CD4_EM_, higher PD-1^NEG^Tim-3^+^CD8^+^, and more CTLA-4^NEG^ T_reg_ at baseline survived longer. Furthermore, an increase in Tim-3^+^ natural killer cells following vaccination conferred survival benefits (78). Similarly, Klyushnenkova et al. (79) illustrated the importance of breaking immunological tolerance to “self” antigen by targeting CD25^+^ T_reg_ during CTLA-4 blockade in mice. Although this research has several shortcomings, it highlights the potential value of such combination therapies.

**Table 2:** **Prostate cancer and immunotherapy**.

Therapeutic vaccines	Description	Response	Status
Sipuleucel-T (Provenge) (72, 77)	Dendritic cell vaccine pulsed with a chimeric protein expressing GM-CSF and PAP as a tumor-associated antigen	Correlation found between PAP antibody titer and patient survival, but not with T-cell proliferative response to PAP (74)	FDA Approved 2010 for metastatic CRPC patients
			Clinical trial showed a relative 22% reduction in risk of death and 4.1-month improvement in median survival as compared with placebo group. Despite improvement in overall survival (OS), no difference in progression-free survival (PFS) was observed (72)
			Phase II trial with or without radiation in patients with metastatic CRPC
			Phase II trial with enzalutamide in patients with metastatic CRPC
			Phase II trial with indoximod, an IDO pathway inhibitor for patients with refractory metastatic disease
			Phase II trial with or without pTVG-HP, a DNA-based vaccine expressing PAP
			Studies evaluating combination with ipilimumab (2)
PROSTVAC-VF (93)	PSA Antigen + 3 costimulatory molecules delivered in viral vectors		Phase III trial started in 2011 in metastatic CRPC patients
			Phase II trial in minimally symptomatic CRPC patients showed no difference in PFS, but demonstrated an 8.5-month improvement in median OS and a 44% reduction in risk of death (2, 73)
			Phase II study in combination with samarium-153 IV radiation in patients with CRPC and bone metastasis (2)
			Phase II trial in active surveillance patients ongoing
			Two Phase II trials in combination with ipilimumab (2)
GVAX (76)	Irradiated prostate cancer cell lines (LNCaP and PC3) engineered to express GM-CSF (75, 76)	Only antibody-mediated responses to vaccine were detected (74)	Phase III trials terminated for lack of success (2, 74)
			Phase I/II trial of GVAX + hormone therapy
			Two Phase III trials in combination with CTLA-4 inhibitor in chemotherapy naïve and chemotherapy-experienced CRPC patients (74)
			PSMA seroreactivity is a potential biomarker for efficacy of GVAX + ipilimumab (73)
NCT00583752	Adenovirus/PSA vaccine in men with recurrent PCa after local therapy	Pending	Phase I trail confirmed safety
			Phase II trial in men with accrual almost completed (2)
			Protocol: vaccine alone or ADT followed by vaccinations 14 days later
NCT00583024	Adenovirus/PSA vaccine in men with hormone-refractory PCa	Pending	Phase II trial with accrual completed but results pending
NCT01875250 (94)	Enzalutamide alone or in combination with PROSTVAC/TRICOM in non-metastatic hormone sensitive PCa patients	Pending	Phase II study ongoing
DNA-PAP and DNA-PSA (2)	DNA-based vaccines	Two of three patients in highest dosing group saw increased PSA doubling time and increased levels of PSA-specific IFN-γ-producing T lymphocytes	Phase I trials demonstrated safety in humans
ProstAtak	Adenoviral vector expressing Herpes virus thymidine kinase targets tumor cells and is followed by anti-herpes drug valacyclovir (Valtrex)		Phase III trial with radiation for patients with localized prostate cancer

**Checkpoint inhibitors/immune modulators**	**Description**		**Status**

Ipilimumab (Yervoy)	CTLA-4 checkpoint inhibitor		Phase III trial combined with low-dose radiation in late stage mCRPC patients treated with docetaxel was negative, but showed a trend toward improved survival (95)
			Phase III trial in chemotherapy naïve mCRPC patients (NCT01057810) has completed accrual but results are not available
			Phase II trial following Provenge for patients with chemo-naïve CRPC
			Phase II trial plus ADT for patients with incomplete response to ADT alone for patients with metastatic CRPC
			Phase II trial in combination with hormonal therapy, degarelix, and radical prostectomy in men with newly diagnosed metastatic castration sensitive disease or degarelix in patients with biochemically recurrent metastatic castrate sensitive disease after radical prostectomy
CT-011	Anti-PD-1 antibody		Phase II study with cyclophosphamide for advanced prostate cancer
	OX40 antibody		Phase I/II trial for patients with metastatic prostate cancer who have failed prior ADT + docetaxel
Lirilumab	Anti-KIR antibody		Phase I trial in combination with nivolumab (anti-PD-1) in advanced solid tumors
MSB0010718C	Anti-PD-1		Phase I trial in patients with solid tumors

**Adoptive cell therapy**	**Description**		**Status**

	T cells genetically engineered to target cancer specific antigen NY-ESO-1		Phase II trail following preparative chemotherapy regimen
	T cells genetically engineered to target cancer specific antigen NY-ESO-1		Phase II study in combination with dendritic cell based vaccine also using NY-ESO-1

**Monoclonal antibodies**	**Description**		**Status**

Rituximab	Monoclonal antibody targeting CD20		Phase 0 trial as neoadjuvant therapy in patients scheduled to undergo radical prostatectomy

## Summary

The presence of inflammatory cells in the prostate is well documented. Evidence suggests that these cells may drive PCa development. Additionally, growing tumors can induce recruitment of immune cells into the prostate microenvironment and initiate a reciprocal interaction that promotes disease progression. Surprisingly, information regarding precise profiles of prostate tumor-infiltrating leukocytes is extremely limited. Individual cells and the overall immune response have the potential to be anti- or pro-tumorigenic, depending on cellular phenotypes, combinations, localization and the tumor microenvironment. Thus, the identities, traits, functional status, distribution, kinetics, and interactions of inflammatory cells in the prostate and PCa must be fully characterized in order to elevate and combine the already promising current immunotherapies. Furthermore, efforts must be directed at elucidating better indicators of potential response to facilitate identification of ideal target populations, as well as biomarkers to assess therapeutic efficacy. Progress in these areas will strengthen the existing optimism that immunotherapy will become part of a successful standard care regimen for future PCa patients.

## Conflict of Interest Statement

The authors declare that the research was conducted in the absence of any commercial or financial relationships that could be construed as a potential conflict of interest.
